# NOD2 Deficiency Promotes Intestinal CD4+ T Lymphocyte Imbalance, Metainflammation, and Aggravates Type 2 Diabetes in Murine Model

**DOI:** 10.3389/fimmu.2020.01265

**Published:** 2020-07-07

**Authors:** Daniela Carlos, Malena M. Pérez, Jefferson A. Leite, Fernanda A. Rocha, Larissa M. S. Martins, Camila A. Pereira, Thais F. C. Fraga-Silva, Taís A. Pucci, Simone G. Ramos, Niels O. S. Câmara, Vânia L. D. Bonato, Rita C. Tostes, João S. Silva

**Affiliations:** ^1^Departments of Biochemistry and Immunology, University of São Paulo, Ribeirão Preto, Brazil; ^2^Pharmacology, University of São Paulo, Ribeirão Preto, Brazil; ^3^Pathology and Legal Medicine, Ribeirão Preto Medical School, University of São Paulo, Ribeirão Preto, Brazil; ^4^Department of Immunology, Institute of Biomedical Science (ICB), University of São Paulo, Ribeirão Preto, Brazil; ^5^Fiocruz-Bi-Institutional Translational Medicine Plataform, Ribeirão Preto, Brazil

**Keywords:** Innate immunity receptor, helper T lymphocytes, metainflammation, gut microbiota, obesity and type 2 diabetes

## Abstract

Type 2 diabetes (T2D) is a metabolic disease characterized by increased inflammation, NOD-like receptors (NLRs) activation and gut dysbiosis. Our research group has recently reported that intestinal Th17 response limits gut dysbiosis and LPS translocation to visceral adipose tissue (VAT), protecting against metabolic syndrome. However, whether NOD2 receptor contributes intestinal Th17 immunity, modulates dysbiosis-driven metabolic tissue inflammation, and obesity-induced T2D remain poorly understood. In this context, we observed that mice lacking NOD2 fed a high-fat diet (HFD) display severe obesity, exhibit greater adiposity, and more hepatic steatosis compared to HFD-fed wild-type (WT) mice. In addition, they develop increased hyperglycemia, worsening of glucose intolerance, and insulin resistance. Notably, the deficiency of NOD2 causes a deviation from M2 macrophage and regulatory T cells (Treg) to M1 macrophage and mast cells into VAT compared to WT mice fed HFD. An imbalance was also observed in Th17/Th1 cell populations, with reduced IL-17 and IL-22 gene expression in the mesenteric lymph nodes (MLNs) and ileum, respectively, of NOD2-deficient mice fed HFD. 16S rRNA sequencing indicates lower richness, alpha diversity, and a depletion of *Allobaculum, Lactobacillus*, and enrichment with *Bacteroides* genera in these mice compared to HFD-fed WT mice. These alterations were associated with disrupted tight-junctions expression, augmented serum LPS, and bacterial translocation into VAT. Overall, NOD2 activation is required for a protective Th17 over Th1 immunity in the gut, which seems to decrease gram-negative bacteria outgrowth in gut microbiota, attenuating the endotoxemia, metainflammation, and protecting against obesity-induced T2D.

## Introduction

T2D is a chronic inflammatory disease characterized by hyporesponsiveness to insulin and glucose intolerance resulting in alterations of β-cell function, structure, or both. The etiology of T2D is multifactorial and linked to genetic, environmental, dietary, and metabolic factors. Obesity is considered a major cause of insulin resistance development and subsequent T2D (1). Interestingly, many insulin resistant individuals do not develop T2D because, in some cases, the β cells compensate the deficit of insulin responsiveness by increasing insulin secretion. Thus, only 1/3 of obese individuals develop chronic hyperglycemia and T2D. The reasons for this heterogeneity are not yet fully understood, although genetic, epigenetic, and environmental factors seem to be involved (2).

Obesity correlates with the development of an inflammatory process in the adipose tissue that results from activation of innate immune cells in response to nutrients excess or microbial products. Recent studies have demonstrated a differential gene expression profile in macrophages recruited to adipose tissue in non-obese and HFD-fed obese mice. In particular, marked proinflammatory M1 macrophages linked to tumor necrosis factor-α (TNF-α) and inducible nitric oxide synthase (iNOS) expression occurs in obese mice, whereas resident macrophages in non-obese mice exhibit a M2 phenotype linked to IL-10 and arginase-1 expression (3). It was proposed that obesity progression shifts macrophage phenotype (to the M1 over the M2 phenotype) in adipose tissue, contributing to the development of insulin resistance and T2D. Mast cells also accumulate in the subcutaneous adipose tissue of obese mice, producing IL-6, and interferon gamma (IFN-γ), which promote apoptosis and angiogenesis during T2D (4). In addition, monocytes from diabetic patients synthesize several cytokines such as IL-6, IL-8, TNF-α, and IL-1β (5).

Although several studies mainly emphasize the importance of myeloid cells, recent observations demonstrate that lymphocytes are also involved in the induction and regulation of obesity-induced T2D. In general, IFN-γ-producing lymphocytes (Th1) cause insulin resistance whereas Th2 lymphocytes tend to counteregulate this response (6). In addition, decreased regulatory T cells (Treg) in adipose tissue of obesity and T2D experimental models has been reported (7). More recently, increased Th17 lymphocytes and reduced Treg cells in the circulation of diabetic patients were associated with T2D progression (8). A causal interaction between the gut microbiota and obesity has been reported in “germ-free” mice colonized with the gut microbiota of ob/ob obese mice. These mice gain more weight compared to mice that receive gut microbiota of non-obese mice (9), implying that the microbiota is involved in the control of energy metabolism. In fact, metagenomic studies showed increased levels of Firmicutes in obese subjects and mice, which correlate with a profile of genes encoding enzymes that degrade non-degraded polysaccharides, favoring extra energy harvesting from the diet.

An additional mechanism by which the microbiome contributes to metabolic disorders appears to be by initiating a systemic inflammation. Intestinal phagocytes, such as dendritic cells and macrophages, capture intestinal bacterial antigens through a process known as bacterial translocation, causing a “low grade bacteremia” mediated by both CD14 and NOD1 receptors in diabetic mice (10). A more recent study showed that the gut microbiota *per se* can counteract a genetically determined condition in mice lacking TLR2 that predisposes to the T2D phenotype (11). Taken together, NOD2 receptor activation contributes to intestinal Th17 response, which can limit gut microbiota dysbiosis and disruption of intestinal barrier. In turn, these mechanisms reduce LPS translocation to the VAT, attenuate metainflammation and obesity-induced T2D.

## Materials and Methods

### Mice and Experimental Groups

Nod2^−/+^ mice backcrossed on a C57BL/6 background were obtained from the Congenics Facility at Yale University (kindly provided by Dr. Richard Flavell, Yale University) and bred with C57BL/6 mice to establish a Nod2^−/−^ colony (12). Female, 4–6 weeks-old, NOD2 deficient (NOD2^−/−^), and C57BL/6 controls were used. Mice were kept in the animal house of the Department of Biochemistry and Immunology, FMRP-USP, where they were provided filtered air and free access to water and food. Mice were reared under specific pathogen–free conditions. The experiments were carried out in accordance with the National Council for Animal Experimentation Control (CONCEA) and were approved by the Ethics Committee on Animal Use (CEUA) of the University of Sao Paulo, Ribeirao Preto, Brazil (protocol number 144/2014). The mice were divided into group I, WT mice fed a control diet (CTD-AIN 93, comprising 9.7% fat, 77.1% carbohydrate, and 13.4% protein); Group II, NOD2^−/−^ mice fed the CTD; group III, WT mice fed a high-fat diet (HFD-D12492, comprising 60% fat, 20% carbohydrate, and 20% protein) and group IV, NOD2^−/−^ mice fed the HFD. C57BL/6 and NOD2^−/−^ mice were fed the control diet or HFD for 20 weeks. During this period, nutritional, metabolic, and immunological parameters were analyzed.

### Nutritional Parameters

The nutritional profile was determined by analyzing food intake, body weight, visceral (mesenteric) fat mass, total fat mass, and adiposity index. Body weight of mice was measured weekly, using a digital scale. The amount of total fat mass was determined by the sum of deposits of retroperitoneal and mesenteric fats. The adiposity index was calculated by dividing the total body fat by the final body weight, multiplied by 100.

### Metabolic Parameters

For the glucose tolerance test (GTT), mice were submitted to a 12-h fasting period. Blood samples were taken at baseline and after intraperitoneal administration of a solution containing 25% glucose (Sigma-Aldrich, cat. G8270) equivalent to 2.0 g/kg, being collected at 0, 15, 30, 60, and 120 min (min). The ACCU-CHEK®Active equipment was used to read glucose levels. For the insulin tolerance test (ITT), mice were submitted to a 6-h fasting period. Blood samples were taken from mice at baseline and after intraperitoneal administration of regular insulin equivalent to 1.5 IU/kg, being collected at 0, 5, 10, 15, 20, 15, and 30 min.

### Experimental Sampling and Reprodutibility

Three series of experiments, with 3–5 mice in each experimental group, were performed for each specific protocol. In each series of experiments (one representative experiment), 3–5 mice per experimental group were used and samples from these 3–5 mice in each group were separated for specific protocols. VAT samples from one series of experiments e.g., were used either for gene expression analysis or CFU quantification; additional VAT samples from another series of experiments were again used for gene expression analysis or CFU quantification. Therefore, unless otherwise stated, the total sample number in each experimental protocol reflects the number of mice used in the three series of experiments and is described in the figure legends.

### Detection of Total Cholesterol, Triglyceride, and LPS Levels

Mice were fasted for 12 hours (h) and blood was collected by the tip of the tail vein. Hemolysis-free serum was collected after centrifugation. Total cholesterol (Labtest, cat. MS10009010026) and triglyceride (Labtest, cat. MS10009010070) concentrations were measured using kits from Labtest or LPS (Lonza, cat. 50-650U) concentrations using kits from Lonza.

### Quantification of Serum Insulin Levels

Insulin concentrations were determined using the Mouse Ultrasensitive Insulin kit (Alpco Diagnostics, cat. 80-INSMSU-E01) according to the manufacturer's instructions.

### Histopathology and Immunohistochemistry Analysis

Histopathological evaluations of the pancreas, VAT and liver were performed in samples that were fixed in PBS/10% formaldehyde (Sigma-Aldrich, cat. F8775) and stained with hematoxylin and eosin (HE). The material was sectioned, mounted on glass slides and kept in a dry oven at 60°C for 1 h. Then, the material was hydrated and deparaffinized using xylene, alcohol, and water. Immunohistochemistry reactions were performed as previously described (13).

### Evaluation of Bacterial Translocation

To evaluate bacterial translocation into the blood and VAT, samples were aseptically collected. Subsequently, 50 μL aliquots were spread with a sterile loop in Brain Heart Infusion (BHI) medium (Sigma-Aldrich, cat. 53286) containing agar plates and placed in an incubator at 37°C for 48 h to count the colony- forming units (CFU).

### RNA Extraction and Quantitative Real-Time PCR

Total RNA was extracted from the ileum using Trizol (Life Technologies, Molecular Probes, Carlsbad, CA, USA, cat. 15596026) following the manufacturer's instructions. cDNA was obtained using a High Capacity reverse transcription kit (Applied Biosystems, Foster City, CA, USA, cat. 4368814) following DNase treatment (Life Technologies, cat. 18068015). Gene expression was analyzed by qPCR using the SYBR Green PCR Master Mix (Applied Biosystems, cat. 4344963). Specific mRNA expression levels were normalized relatively to β-actin mRNA levels using the comparative 2ΔΔCt method.

### Analysis of Leukocytes by Flow Cytometry

Total mesenteric lymph nodes (MLNs) were removed, placed in plates containing RPMI medium (Corning, cat. 10-040) and macerated using a cell strainer to obtain a cell suspension. The cell suspension was centrifuged (400 g; 10 min) and resuspended in 1 mL of RPMI medium. Flow cytometry analysis was performed on leukocyte suspension with 1 × 10^6^ cells/tube in 100 μL of PBS. First, cell suspensions were incubated with 5% normal rabbit serum for 30 min to block non-specific binding. Next, anti-mouse antibodies against CD3, CD4, CD127, CD90.2 and their control isotypes (BD Pharmingen, San Diego, CA, USA) were added and incubated for 30 min in the dark. Then, the cells were washed and resuspended in FACS Permeabilizing solution (BD Pharmingen, cat. 561651) for 10 min. Next, the expression of transcription factors was assessed by incubating cells with antibodies against T-bet, Foxp3, or ROR-γt (BD Pharmingen). IL-17 expression was evaluated after *in vitro* re-activation with phorbol myristate acetate (PMA, 25 ng/ml) (Sigma-Aldrich, cat. P8139) and ionomycin (1 mg/ml, Sigma–Aldrich, cat. 10634) plus monensin 10 mg/ml (Sigma–Aldrich, cat. 46468). The cells were analyzed using a FACS Canto flow cytometer, and the data were analyzed using FlowJo (Tree Star) software. The following antibodies were used: CD3-APC-C7: Catalog (557596) Clone (145-2C11); CD4-PercP: Catalog (553052) Clone (RM4-5), CD127-PECy7: Catalog (560733) Clone (5B/199), CD90.2-APC-Cy7 (561641) Clone (53-2.1), T-bet-Alexa 647: Catalog (644813) Clone (4B10), Foxp3-PE: Catalog (560408) Clone (MF23), IL-17-PE: Catalog (559502), and ROR-gt-Alexa Fluor 647: Catalog (562683) Clone (Q-31-378).

### Western Blotting

Insulin was administrated intraperitoneally (3.8 IU/kg body weight) and after 5 min the gastrocnemius skeletal muscle was collected. The fragments were homogenized with a polytron in extraction buffer: Tris 0.05 mol/L (Sigma-Aldrich, cat. 10708976001) sodium chloride (NaCl) 0.150 mol/L (Sigma-Aldrich, cat. 55886), ethylenediaminetetraacetic acid (EDTA) 0.001 mol/L (Sigma-Aldrich, cat. E9884), Triton X-100 1% (Sigma-Aldrich, cat. X100), deoxycholate 1% (Sigma-Aldrich, cat. D6750), sodium dodecyl sulfate (SDS) 0.1% (Sigma-Aldrich, cat., 436143), sodium orthovanadate (Na_3_VO_4_) 0.001 mol/L - 1: 100 (Sigma-Aldrich, cat.S6508), sodium fluoride (NaF) 0.01 mol/L - 1: 100 (Sigma-Aldrich, cat. 201154) and protease inhibitor (Thermofisher, cat. 78429). Fifty micrograms of extracted proteins were loaded directly into SDS sample buffer for 10% SDS-polyacrylamide gel electrophoresis. After transferring the samples onto a nitrocellulose membrane (Trans-Blot Transfer Medium; Bio-Rad, Hercules, CA, cat. 1704156), the membranes were blocked with 5% milk in Tris buffer solution containing 0.1% Tween 20 (Sigma-Aldrich, cat. P1379) for 1 h and then incubated overnight at 4°C with antibodies against GLUT-4 (Cell Signaling, cat. 2299), pSer^473^-Akt (Cell Signaling, cat. 9271), or total AKT (Cell Signaling, cat. 9272S), at 1: 500 dilution. Next, it was were incubated with an enzyme horseradish peroxidase (HRP)-conjugated secondary Ab (Cell Signaling, cat. 7076S) for 1 h at room temperature. After the membranes were rinsed, the immunocomplexes were developed using an enhanced peroxidase/luminol chemiluminescence reaction ECL Western blotting detection reagents (Pierce Biotechnology, cat. 32109) and exposed to X-ray film with autoradiography (Carestream Health). The intensity of the bands was evaluated by densitometric analysis using ImageJ software.

### 16S rRNA Gene Sequencing

16S rRNA gene sequencing was performed, as previously described (14). The Ribosomal Database Project classifier (RDP) and the May 2013 Green genes taxonomy were used to assign taxonomy to representative operational taxonomic units (OTUs) (15). A quantitative analysis on the richness, evenness and Shannon diversity, defined as alpha diversity, indices of the gut microbiome were calculated using PAST 3.26 software (16). The gut microbiota analysis is representative of a single experiment (*n* = 3–5 mice per group). Our results indicate that gut microbiota alterations are linked to the obesity-induced type 2 diabetes phenotype. However, due to the small sample size, additional studies are necessary to bring conclusive results.

### Statistical Analysis

Data are expressed as mean ± standard error of the mean (SEM). The differences observed among the several experimental groups were analyzed by one-way ANOVA followed by the parametric Tukey test for comparing multiple groups. All analyses were performed using Prism 5.0 software (GraphPad Software). Statistical significance was set at *P* < 0.05.

## Results

### NOD2 Deficiency Aggravates Hfd-Induced Obesity Development and Metabolic Inflammation

**Figure 1:**
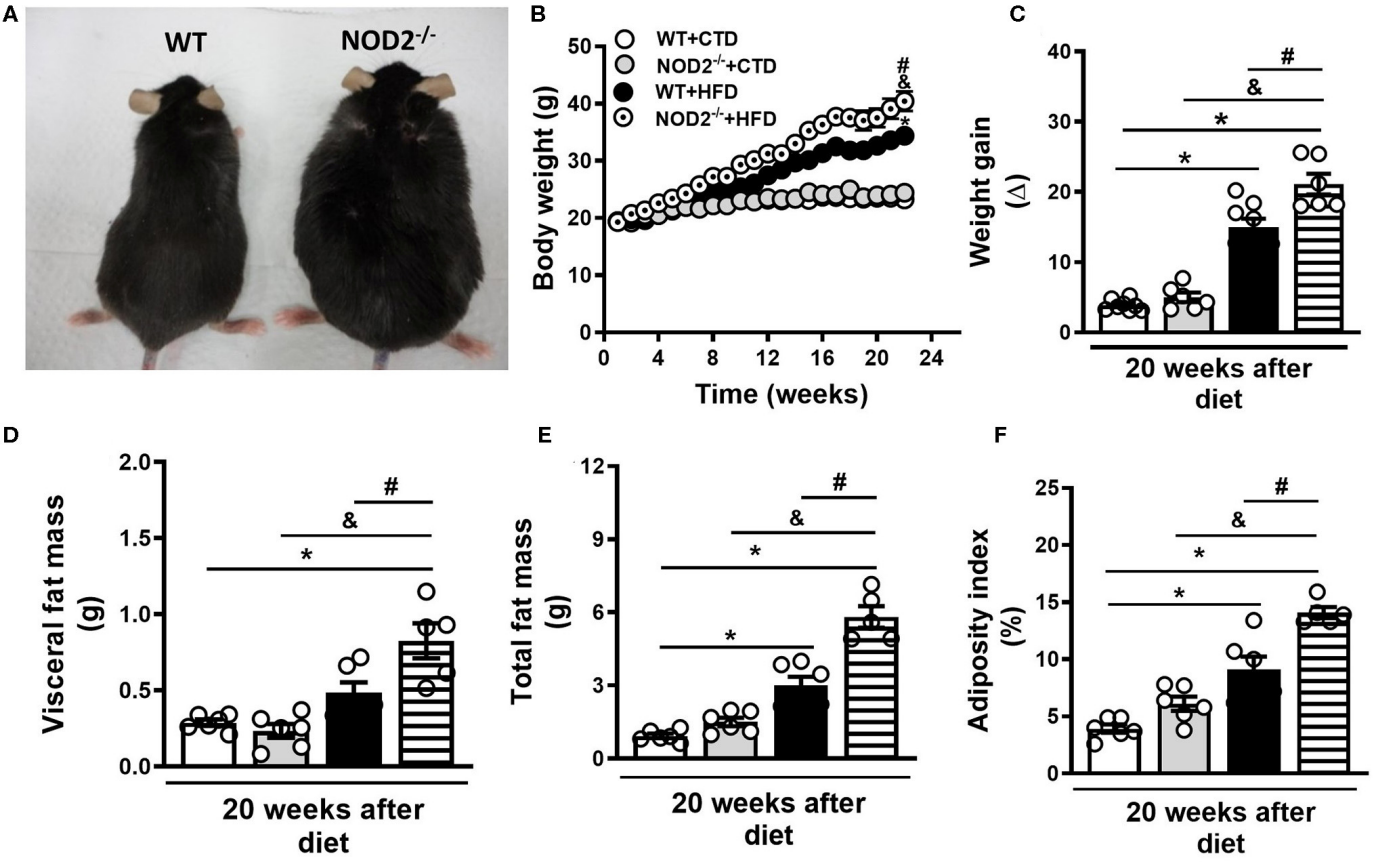
Nutritional parameters of WT and NOD2^−/−^ mice fed a CTD or a HFD. Representative images of NOD2^−/−^ mice (right) and WT mice (left) fed a HFD **(A)**. Body weight **(B)**, weight gain **(C)**, VAT mass **(D)**, total fat mass **(E)**, and adiposity index **(F)** were determined in NOD2^−/−^ and WT mice after 20 weeks on HFD or CTD. The results are expressed as the mean ± SEM and are a compilation of 3 independent experiments (*n* = 5–8 mice per group). Asterisks represent statistically significant differences (*p < 0.05) compared to WT on CTD; (^#^*p* < 0.05) compared to WT on HFD; (^&^*p* < 0.05) compared to NOD2^−/−^ mice on CTD. Significant differences between the groups were compared by one-way ANOVA followed by Tukey's multiple-comparison test.

**Figure 2:**
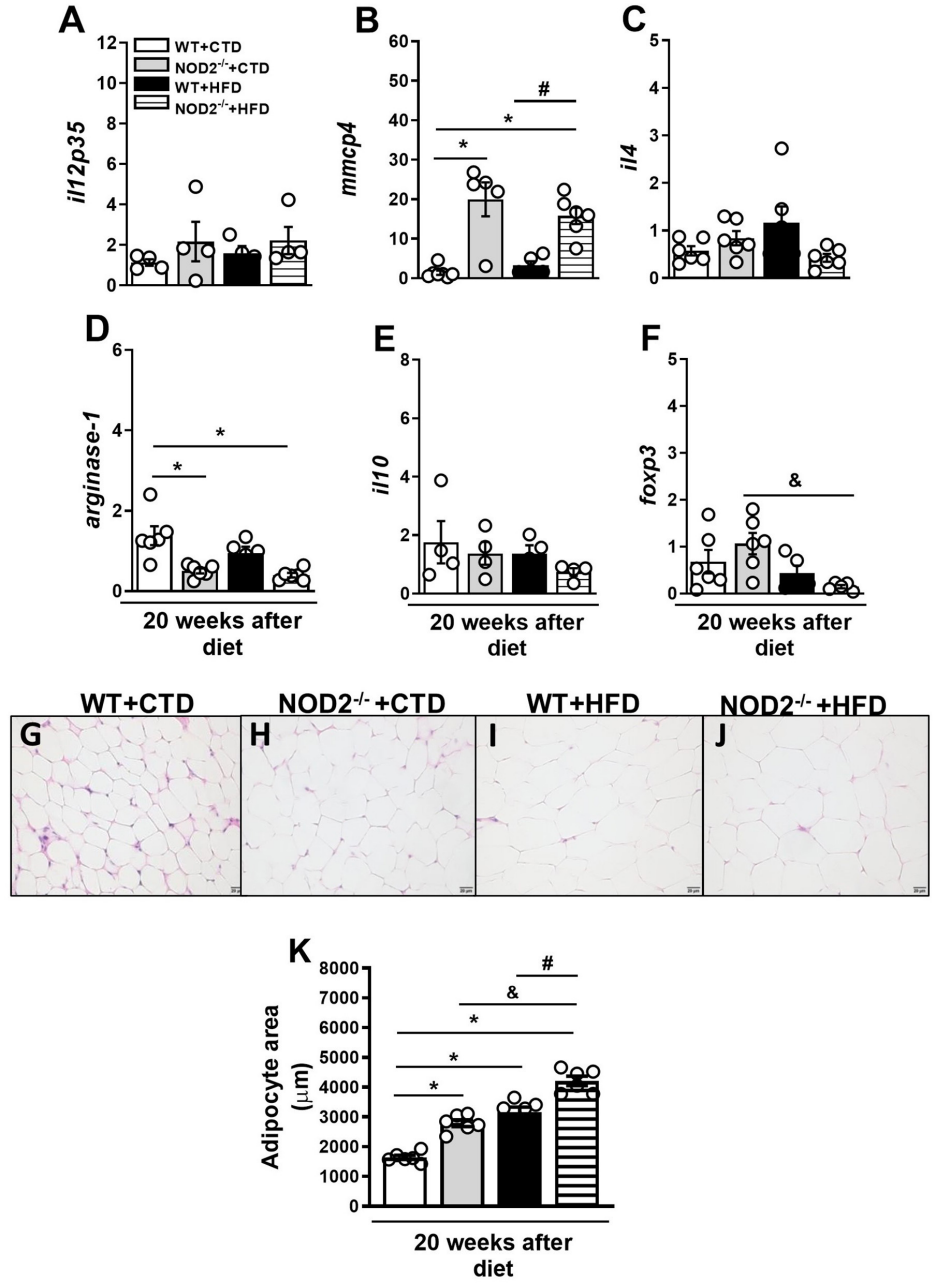
Gene expression profile of pro- and anti-inflammatory molecules in VAT of WT and NOD2^−/−^ mice fed a CTD or HFD. Relative expression of IL-12p35 **(A)**, mMCP-4 **(B)**, IL-4 **(C)**, arginase-1 **(D)**, IL-10 **(E)**, and Foxp3 **(F)** in VAT by RT-PCR. Adipocyte hypertrophy was assessed in NOD2^−/−^ and WT mice after 20 weeks on HFD or CTD **(G–J)** (original magnification 400x). **(K)** Morphometric quantification of adipocyte size in VAT of WT and NOD2^−/−^ mice fed a CTD or HFD. The results are expressed as the mean ± SEM and are a compilation of 3 independent experiments (*n* = 4–6 per group). Asterisks represent statistically significant differences (*p < 0.05) compared to WT on CTD; (^#^*p* < 0.05) compared to WT on HFD; (^&^*p* < 0.05) compared to NOD2^−/−^ mice on CTD. Significant differences between the groups were compared by one-way ANOVA followed by Tukey's multiple-comparison test.

Initially, we evaluated the nutritional profile of NOD2^−/−^ and WT mice fed a high-fat diet (HFD) or a control diet (CTD) for 20 weeks. As shown in Figure 1A, under a HFD, NOD2^−/−^ mice were more prone to develop obesity compared to WT mice. NOD2^−/−^ mice exhibited increased body weight, starting after 10 weeks, and significant increased weight gain compared to WT mice at 20 weeks after HFD (Figures 1B,C). Although there were no differences in food intake (data not shown), NOD2^−/−^ mice displayed augmented visceral and total fat accumulation and an increased adiposity index compared to WT mice under the HFD (Figures 1D,F). In addition, NOD2^−/−^ mice fed HFD also exhibited increased expression of IL-12p35 (M1 macrophage marker) (Figure 2A) and mMCP-4 (mast cell marker) (Figure 2B) associated with decreased IL-4 and arginase-1 expression (M2 macrophage markers) compared to WT mice on HFD or CTD, respectively (Figures 2C,D). Interestingly, we observed a significant decline in Foxp3 expression (regulatory T cell marker) in VAT of NOD2^−/−^ mice fed HFD compared to NOD2^−/−^ mice fed CTD (Figure 2F). Despite a trend to decreased IL-10 expression in the VAT, NOD2 deficiency did not significantly affect IL-10 levels in comparison the to other experimental groups (Figure 2E). Moderate adipocyte hypertrophy was observed in CTD-fed NOD2^−/−^ mice compared to WT mice fed CTD (Figures 2G,H). However, under the HFD, adipocytes of the NOD2^−/−^ mice exhibited greater hypertrophy compared to WT mice (Figures 2I,J). In fact, the quantitative analysis revealed that NOD2 deficiency significantly increased adipocyte size in VAT compared to WT mice fed HFD (Figures 2I-K). These findings demonstrate that NOD2 receptor mitigates obesity development and metainflammation in a HFD-induced experimental model.

### NOD2 Deficiency Exacerbates Lipid and Glucose Metabolism Alterations and Confers Susceptibility to T2D Development

**Figure 3:**
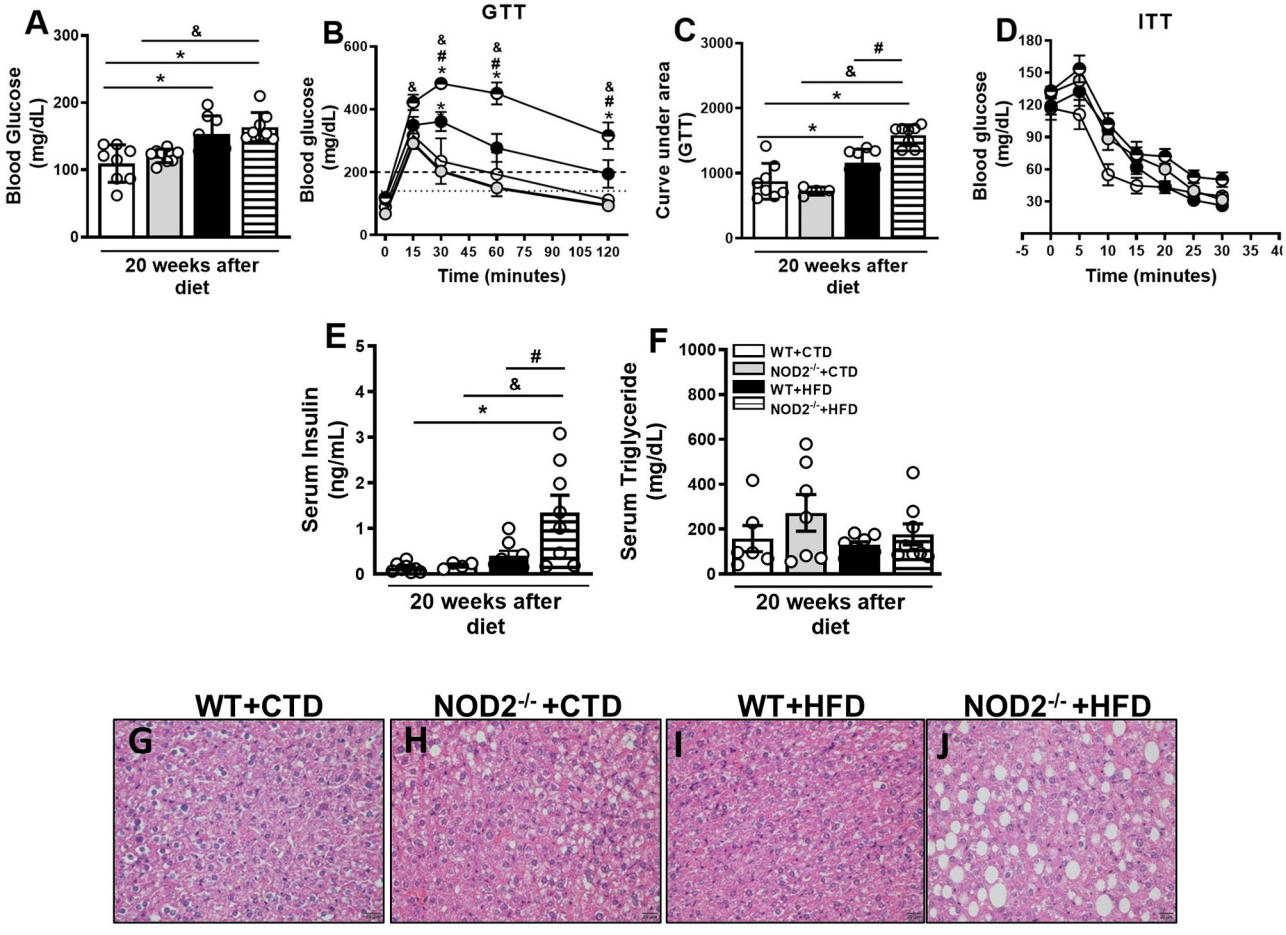
Metabolic parameters and fat deposition in liver of WT and NOD2^−/−^ mice fed a CTD or HFD. Fasting blood glucose levels **(A)**, and glucose levels after glucose tolerance test (GTT) **(B)**, area under the curve for the GTT **(C)** and insulin tolerance test (ITT) **(D)**. Fasting insulin **(E)** or triglyceride **(F)** levels were determined in the serum. Hepatic steatosis was assessed in NOD2^−/−^ and WT mice after 20 weeks on HFD or CTD **(G–J)** (original magnification 400x). The results are expressed as the mean ± SEM and are a compilation of 3 independent experiments (*n* = 4–8 per group). Asterisks represent statistically significant differences (*p < 0.05) compared to WT on CTD; (^#^*p* < 0.05) compared to WT on HFD; (^&^*p* < 0.05) compared to IL-23p19^−/−^ mice on CTD. Significant differences between the groups were compared by one-way ANOVA followed by Tukey's multiple-comparison test.

Next, we investigated parameters related to glucose metabolism, such as fasting glycemia, glucose intolerance, and insulin resistance. In addition to worsening of obesity, NOD2^−/−^ mice exhibited increased fasting blood glucose levels (Figure 3A). NOD2^−/−^ mice were also more intolerant to glucose. Likewise NOD2^−/−^ mice became diabetic since they displayed glucose levels above 200 mg/dL 2 h after the GTT (Figures 3B,C). On the other hand, HFD-fed WT mice were in a pre-diabetic state since glucoce levels were in the 149–200 mg/dL range 2 h after the GTT (Figures 3B,C). HFD-fed NOD2^−/−^ mice also demonstrated more insulin resistance, as shown by the ITT test, and higher levels of serum insulin in comparison to WT mice on the HFD (Figures 3D,E), confirming a state of hyperinsulinemia. During obesity and insulin resistance, decreased lipogenesis, and increased lipolysis and, consequently, marked degradation of triglycerides and inappropriate release of circulating fatty acids are observed. In agreement, under the HFD, NOD2^−/−^ mice exhibited a trend to increased serum triglyceride, but not cholesterol levels compared to WT mice (Figure 3F and data not shown). In addition, NOD2 deficiency caused a slight fat deposition in the liver, i.e., hepatic steatosis, in CTD-fed mice compared to WT mice on CTD (Figures 3G,H). However, HFD increased fat deposition in NOD2^−/−^ mice compared to WT mice (Figures 3I,J). These results indicate that the NOD2 receptor attenuates the metabolic alterations and delays obesity-induced T2D onset.

### NOD2 Deficiency Compromises Insulin Signaling and Pancreatic Islet Function

Insulin regulates glucose homeostasis and its effects rely on activation of the insulin receptor, which occurs by tyrosine autophosphorylation on various substrates, such as the insulin receptor substrate (IRS)-1 and 2. Following tyrosine phosphorylation, IRS-1 and IRS-2 bind and activate PI3 kinase (PI3K), which increases serine phosphorylation of serine/threonine-specific protein kinase (AKT) that, in turn, leads to the transport of glucose via GLUT-4 into skeletal muscle and adipose tissue. Further studies were performed to evaluate intracellular pathways of insulin signaling, such as the expression and phosphorylation of GLUT-4 and AKT in the skeletal muscle. Despite the lack of significancy, the Western blot (WB) analysis showed a decrease in GLUT-4 and total AKT expression in the skeletal muscle of HFD-fed NOD2^−/−^ mice compared to WT mice, indicating a defect in insulin signaling, especially in AKT expression (Supplementary Figures 1A,B,D). However, no significant differences in phosphorylated AKT expression were observed between these mice (Supplementary Figures 1C,D). In addition, NOD2^−/−^ mice fed HFD had increased size of the pancreatic islets, which seems a compensatory mechanism of insulin-expressing β cells expansion (Supplementary Figures 1E–H). In agreement, immunohistochemistry analysis revealed increased staining of insulin-expressing β cells in the pancreatic islets of NOD2^−/−^ mice, confirming a pancreatic hyperplasia state (Supplementary Figures 1I–L). These data demonstrate that NOD2 receptor inhibits insulin resistance, pancreatic islet dysfunction, and retards obesity-induced T2D development.

### NOD2 Deficiency Dampens Th17 Generation and IL-17 Expression in Intestinal Mucosa During T2D

**Figure 4:**
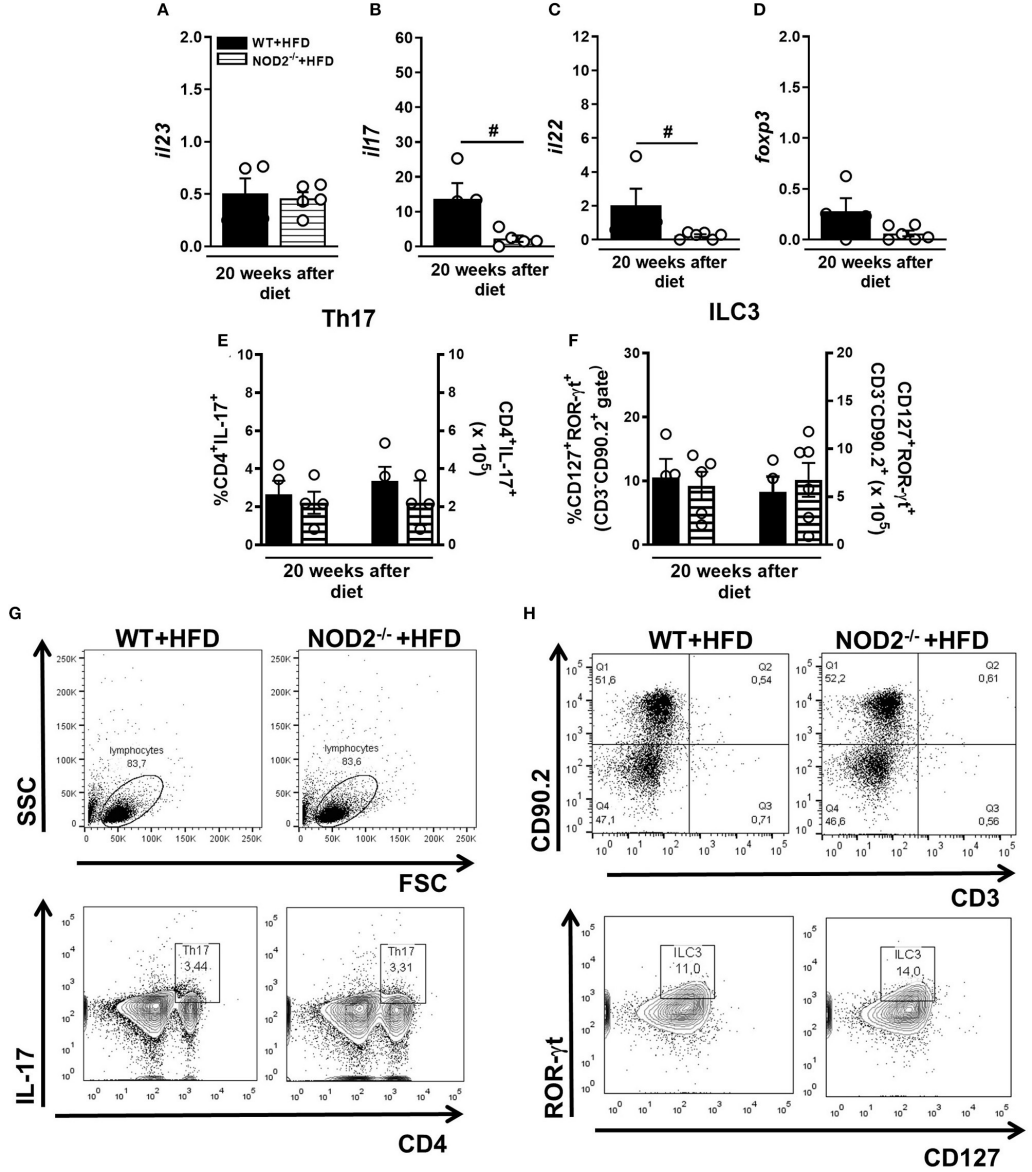
Th17 and ILC3 response generation in MLN and cytokine profile in small intestine of WT and NOD2^−/−^ fed a CTD or HFD. Relative expression of IL-23 **(A)**, IL-17 **(B)**, IL-22 **(C)** and Foxp3 **(D)** was assessed in NOD2^−/−^ and WT mice after 20 weeks on HFD or CTD by RT-PCR. Percentage and absolute numbers of Th17 (CD4^+^IL-17^+^) **(E)** and ILC3 cells (CD127^+^ROR-γt^+^) **(F)** were determined in MLNs by flow cytometry. Percentages of Th17 or ILC3 are shown in representative dot plots in lymphocyte or CD3^−^CD90.2^+^ gates, respectively **(G,H)**. The results are expressed as the mean ± SEM and are a compilation of 3 independent experiments (*n* = 4–6). Asterisks represent statistically significant differences (*p < 0.05) compared to WT on CTD; (^#^*p* < 0.05) compared to WT on HFD; (^&^*p* < 0.05) compared to NOD2^−/−^ mice on CTD. Significant differences between two groups were compared by Student's *t*-test followed Mann-Whitney test; and between more groups by one-way ANOVA followed by Tukey's multiple-comparison test.

Gene expression of IL-23, IL-17, and IL-22 was determined in the small intestine (ileum) of mice fed the HFD. NOD2 deficiency did not alter IL-23 expression in the ileum compared to WT mice on HFD (Figure 4A). However, expression of IL-17 and IL-22 significantly decreased in HFD-fed NOD2^−/−^ mice compared to WT mice on the HFD (Figures 4B,C). No changes in the percentage, but a trend to decreased absolute numbers of Th17 cells (CD3+CD4+IL-17+) in the MLNs of HFD-fed NOD2^−/−^ mice, in comparison to WT mice on the HFD were observed (Figures 4E,G). On the other hand, NOD2^−/−^ mice did not exhibit significant differences neither in the percentage nor in absolute numbers of ILC3 (CD3^−^ CD90.2+ CD127+ROR-γt+) in the MLNs, in comparison to WT mice fed HFD (Figures 4F,H). Therefore, our findings reveal that NOD2 receptor contributes to Th17 generation and cytokine production in the intestinal mucosa during obesity-induced T2D development.

### NOD2 Deficiency Favors Th1 Generation and IFN- γ Expression in Intestinal Mucosa During T2D in Murine Model

**Figure 5:**
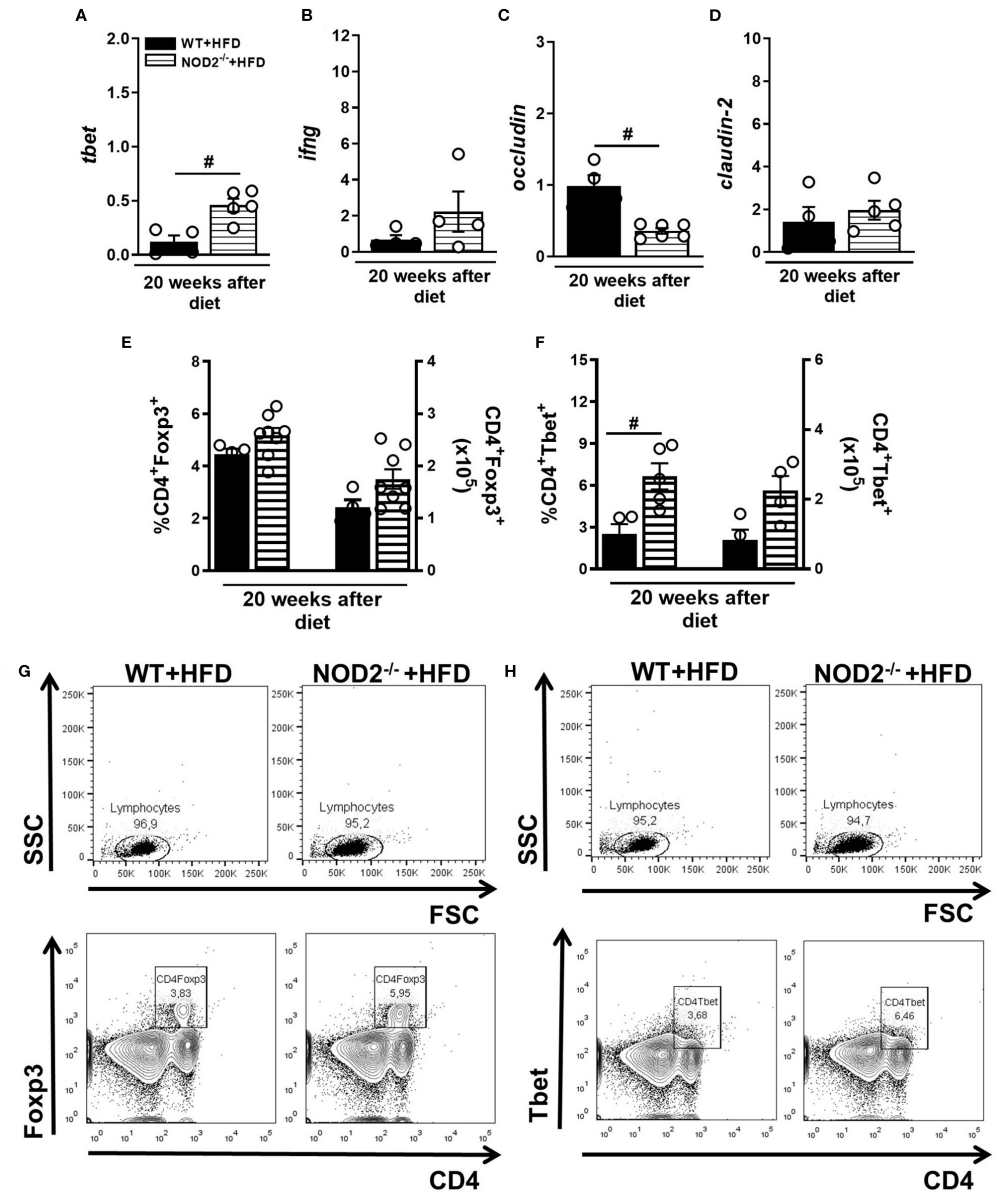
Th1 and Treg response generation in MLN and cytokine profile in small intestine of WT and NOD2^−/−^ mice fed a CTD or HFD. Relative expression of T-bet **(A)**, IFN-γ **(B)**, occludin **(C)** and claudin-2 **(D)** was assessed in NOD2^−/−^ and WT mice after 20 weeks on HFD or CTD by RT-PCR. Percentage and absolute numbers of Treg (CD4^+^Foxp3^+^) **(E)** or Th1 cells (CD4^+^T-bet^+^) **(F)** were determined in MLNs by flow cytometry. Percentages of Treg or Th1 are shown in representative dot plots in lymphocyte gate **(G,H)**. The results are expressed as the mean ± SEM and are a compilation of 3 independent experiments (*n* = 4–8). Asterisks represent statistically significant differences (*p < 0.05) compared to WT on CTD; (^#^*p* < 0.05) compared to WT on HFD; (^&^*p* < 0.05) compared to NOD2^−/−^ mice on CTD. Significant differences between two groups were compared by Student's *t*-test followed Mann-Whitney test; and between more groups by one-way ANOVA followed by Tukey's multiple-comparison test.

In parallel, gene expression of Tbet, IFN-γ, occludin, and claudin-2 was determined in the small intestine (ileum) of mice fed HFD. An increased expression of T-bet and IFN-γ was observed in NOD2^−/−^ mice compared to WT mice on HFD (Figures 5A,B). On the other hand, occludin, but not claudin-2 expression was significantly decreased in the ileum of HFD-fed NOD2^−/−^ mice compared to WT mice on the HFD (Figures 5C,D). Despite an increase, no significant differences were observed in the percentage or absolute numbers of regulatory T cells (Treg) (CD4+Foxp3+) in the MLNs of HFD-fed NOD2^−/−^ mice compared to WT mice on HFD (Figures 5E,G). Foxp3 gene expression was not significantly decreased in the intestinal mucosa of HFD-fed NOD2^−/−^ mice compared to WT mice on the HFD (Figure 4D). Interestingly, the percentage, but not the absolute numbers, of Th1 cells (CD4+Tbet+) was significantly increased in the MLNs of HFD-fed NOD2^−/−^ mice compared to WT mice on HFD (Figures 5F–H). Therefore, our findings infer that NOD2 receptor limits Th1 generation and IFN-γ production in the intestinal mucosa and maintains the gut barrier integrity regulating the expression of tight-junctions' proteins.

### NOD2 Deficiency Alters the Composition of the Gut Microbiota, Increases the Intestinal Permeability, and Metabolic Endotoxemia

**Figure 6:**
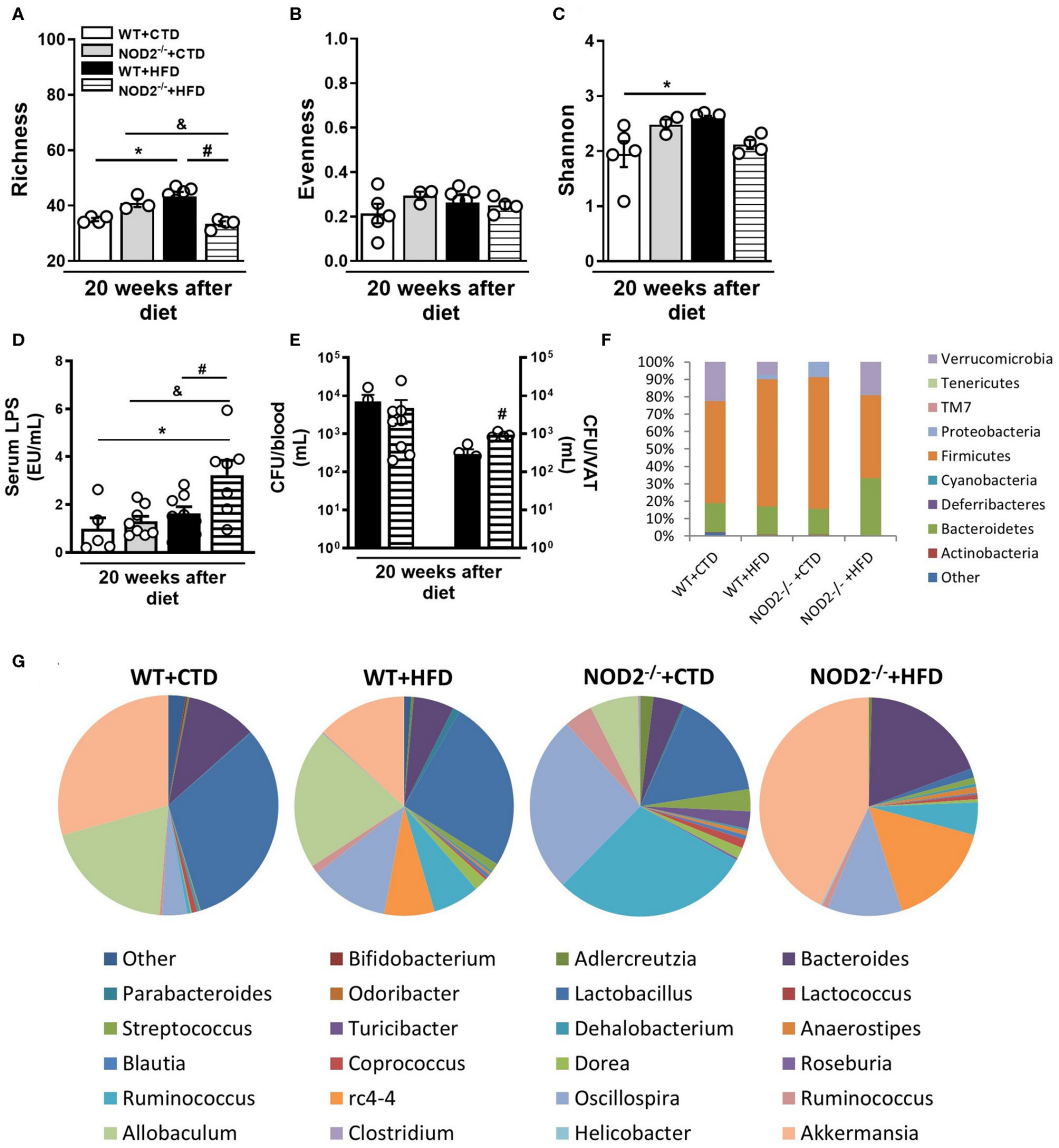
Gut microbiota composition, intestinal permeability and bacterial translocation in WT and NOD2^−/−^ mice fed a CTD or HFD. Richness **(A)**, evenness **(B)**, and Shannon index **(C)** of fecal bacterial OTUs. Serum LPS levels **(D)** and colony-forming unit numbers (CFU) in the blood or VAT **(E)** was assessed. Relative abundance of fecal bacterial phylum **(F)** and genera **(G)** was determined by 16S rRNA gene sequencing. The results are expressed as the mean ± SEM and are a compilation of 3 independent experiments (*n* = 4–8 mice per group) **(D,E)** or represent a single experiment (*n* = 3–5 mice per group) **(A–C,F,G)**. Asterisks represent statistically significant differences (*p < 0.05) compared to WT on CTD; (^#^*p* < 0.05) compared to WT on HFD; (^&^*p* < 0.05) compared to NOD2^−/−^ mice on CTD. Significant differences between two groups were compared by Student's t-test followed Mann-Whitney test; and between more groups by one-way ANOVA followed by Tukey's multiple-comparison test.

The metagenomic analysis of bacterial 16S gene was used to identify OTUs and assess the structure of microbial community through the alpha diversity metrics. Importantly, we did not note differences in richness, evenness, or Shannon-diversity between several experimental groups after 12 weeks of CTD or HFD (**data not shown**). However, the richness, which is the number of OTUs, increased in WT mice fed the HFD in comparison those fed the CTD for 20 weeks (Figure 6A). Additionally, NOD2^−/−^ mice fed the HFD exhibited a significant reduction in the number of OTUs compared to NOD2^−/−^ mice on CTD or to WT mice fed the HFD (Figure 6A). The relative abundance of OTUs, described as evenness, was similar among groups (Figure 6B). The Shannon-diversity index that relates both richness and evenness significantly augmented in WT mice on HFD, but was decreased in NOD2^−/−^ mice fed the HFD compared to WT counterpart mice (Figure 6C).

In order to understand the changes in the microbial community composition, we analyzed the organisms present at different taxonomic levels and their relative abundances. WT mice fed the HFD exhibited an increased abundance of the Firmicutes and Proteobacteria phyla and decreased Verrucomicrobia phylum. CTD-fed NOD2^−/−^ mice, compared to WT fed CTD, exhibited increased abundance of *Firmicutes* and Proteobacteria. Interestingly, NOD2^−/−^ mice on HFD exhibited increased abundance of *Bacteroidetes* and Verrucomicrobia, compared to WT mice on HFD (Figure 6F). Notably, several differences were found at the genera level (Figure 6G). HFD-fed WT mice exhibited higher abundance of *Ruminococcus, Oscillospira*, and rc4-4 associated with lower abundance of *Lactobacillus and Akkermansia* compared to CTD-fed WT mice. Similarly, NOD2^−/−^ mice fed CTD exhibited marked increase of the *Oscillospira* and *Ruminococcus* genera associated with reduced abundance of *Lactobacillus, Allobaculum* and *Akkermansia* genera. Surprisingly, NOD2^−/−^ mice on the HFD exhibited increased abundance of the *Bacteroides*, rc4-4, *Akkermansia* genera and depleted *Lactobacillus* and *Allobaculum* genera (Figure 6E).

Metabolic endotoxemia may account for the metabolic inflammation associated with obesity and T2D. In accordance, our results showed that WT mice fed HFD had a trend to increased LPS levels compared to WT mice on CTD. In addition, NOD2^−/−^ mice fed HFD displayed a significant increase of serum LPS levels compared to NOD2^−/−^ mice fed CTD or WT mice fed HFD (Figure 6D), but without alteration in FITC-Dextran leakage compared to HFD-fed WT mice (**data not shown**). In parallel, NOD2^−/−^ mice exhibited increased CFU counts in the VAT, but not in the blood compared to HFD-fed WT mice (Figure 6E). Taken together, these data show that NOD2 receptor seems to control gut dysbiosis, to reduce metabolic endotoxemia, and VAT translocation, protecting against T2D development.

## Discussion

Obesity and T2D are health problems that affect a considerable number of individuals around the world. It is estimated that by 2,035 more than 592 million people will have diabetes (17). T2D is characterized by low grade inflammation, with increased levels of inflammatory cytokines, along with changes in the gut microbiota. In this context, receptors such as TLRs and NLRs are activated by pathogens or gut microbiota components, which leads to cytokine production that drive the adaptive response. NOD2 receptors, also known as CARD15, recognize the muramyl dipeptide (MDP), the major active component of the peptidoglycan motif, present in gram-negative and gram-positive bacteria. After recognition of their respective ligands, NOD2 self-oligomerizes to recruit and activate the adapter proteins RIP2 and CARD9, resulting in positive regulation of the NF-kB and mitogen-activated protein kinase (MAPK), p38 and c-Jun N-terminal (JNK) signaling pathways, production of inflammatory molecules, and antimicrobial peptides (18, 19).

Our results showed a significant increase in body weight gain and accumulation of visceral and total fat in NOD2 deficient mice fed an HFD for 20 weeks when compared to WT mice, events that were not due to differences in food intake. Our data reinforce previous data showing that NOD2 receptor deficient BALB/c mice fed a HFD have greater propensity to obesity and exhibit key manifestations of metabolic disease compared to WT mice (20). In addition, one study reported that the NOD2 agonist MDP decreased adipose inflammation and glucose intolerance in obese mice, but did not induce changes in the gut microbiome composition (21). Inflammation is one of the factors that contribute to the development of obesity and other metabolic diseases in both human and experimental models (22, 23). During obesity the phenotypic deviation of M2 to M1 macrophages in adipose tissue increases the production of proinflammatory cytokines such as TNF-α and IL-6, which inhibit the insulin receptor signaling (24). Additionally, animals fed a HFD exhibit high levels of mRNA expression coding ICAM-1 and VCAM-1 adhesion molecules, which are responsible for the recruitment of M1 macrophages to adipose tissue during obesity (25). Of fact, our results show intense metainflammation, characterized by increased influx of M1 macrophages and decreased M2 macrophages and Treg cells in VAT of NOD2^−/−^ mice fed a HFD when compared to WT mice. In obese patients, a greater number of mast cells occurs in the VAT, when compared to healthy individuals. In agreement, we observed increased mMCP-4, which is a mast cell specific marker, in the VAT of WT mice, which was more pronounced in NOD2^−/−^ mice on HFD. Mast cells may interact with inflammatory and non-inflammatory cells by direct cell-cell contact or by releasing inflammatory mediators. TNF-α, for example, is an important mediator of mast cells effects and is increased in obese mice. In addition, the neutralization of TNF-α with a soluble TNF receptor attenuates insulin resistance in obese mice (26). Taken together, our data indicate that NOD2 activation limits the adiposity and reduces inflammatory and metabolic defects associated with HFD-induced obesity.

Notably, HFD-fed NOD2^−/−^ mice exhibited a significant reduction of Foxp3, a master transcription factor for Treg cells, in VAT when compared to WT mice on HFD. The MDP dipeptide, which binds to NOD2, reduces Treg apoptosis induced by the Fas ligand. In context, a clear deficiency in the amount of Foxp3^+^ lymphocytes has been reported in mice lacking NOD2 receptors (27, 28). Thus, it is plausible that the reduced Treg cell numbers in VAT of HFD-fed NOD2^−/−^ mice may be a consequence of increased apoptosis of this cell subtype in the VAT. In agreement with the obese phenotype in female HFD-fed NOD2^−/−^ mice, the histological analysis of the VAT showed increased adipocyte size, which reflects adipose tissue hypertrophy. Interestingly, the absence of NOD2 in male mice fed a HFD exacerbates insulin resistance independently of an increase in body mass, promotes bacteria translocation, and inflammation in metabolic tissues (29). The divergent results on insulin resistance and susceptibility to develop obesity may be related to sex differences, since females are more prone to adipose tissue accumulation. Our data add new evidence that NOD2 receptor attenuates migration and activation of inflammatory cells, specially M1 macrophages and mast cells, into VAT, reducing metabolic inflammation and mitigating HFD-induced obesity development.

An important observation is that NOD2^−/−^ mice fed the HFD became diabetic after 20 weeks of HFD, proved by higher levels of blood glucose on GTT test. In addition, a higher concentration of fasting serum insulin was observed in HFD-fed NOD2^−/−^ mice when compared to the other experimental groups. Another interesting fact is that NOD2^−/−^ mice fed an HFD showed fat deposition in the liver. The development of obesity is usually accompanied by disorders in the metabolism of lipids and carbohydrates. In this regard, HFD-fed NOD2^−/−^ mice display a variety of differentially expressed genes primarily related to the intermediate metabolism of lipids and carbohydrates when compared to WT mice fed HFD. Additionally, many of these genes present in NOD2^−/−^ mice have an established role in the development of obesity or are associated with metabolic diseases (30–32). Hepatic steatosis is another very common process in obesity and is considered one of the main causes of the development of insulin resistance (33). Overall, we findings showed that NOD2 activation improves glucose and lipid homeostasis and delays the obesity-induced T2D progression.

We then evaluated the expression of proteins related to glucose uptake and insulin signaling. A trend to decrease in GLUT-4 protein, one of the major glucose transporters in the skeletal muscle of mice, and in total AKT protein was observed in NOD2^−/−^ mice submitted to HFD when compared to HFD-fed WT mice, indicating impaired insulin signaling, which, in turn, can lead to the high levels of blood glucose observed in NOD2^−/−^ mice fed the HFD. As mentioned, NOD2^−/−^ mice developed low-grade inflammation that can inhibit the expression and activity of proteins involved in insulin signaling and glucose transport in the skeletal muscle and adipose tissue. Additionally, we observed alterations in the morphology of pancreatic islets, characterized by hyperplasia and increased insulin expression, of NOD2^−/−^ mice submitted to HFD when compared to HFD-fed WT. This result suggests that in an attempt to lower hyperglycemia, the pancreas produces high amounts of hormones in the early stages of insulin resistance development, subsequently becoming dysfunctional, which completely abolishes insulin production and leads to T2D onset (34). In summary, our data suggest that NOD2 activation alleviates the insulin resistance, pancreatic dysfunction and restricts the obesity-induced T2D onset.

As already described, intestinal homeostasis perturbation and changes in the populations of adaptive and innate immune cells responsible for the control and maintenance of health gut occur during obesity (35, 36). The differentiation of Th17 cells depends on the production of cytokines IL-6, IL-23, and TNF-α by dendritic cells and macrophages induced by microbial factors. Once activated, Th17 cells produce IL-17 and IL-22 cytokines, which play a key role in maintaining the intestinal barrier and modulating the populations of microorganisms present in the gut microbiota (37). In this context a significant reduction in IL-17 and IL-22 gene expression was detected in the ileum of NOD2^−/−^ mice fed the HFD. In parallel, we observed a decrease in Th17 cells in the MLNs of HFD-fed NOD2^−/−^ mice when compared to WT mice. In accordance, NOD2^−/−^ mice have decreased number and impaired activation of Th17 cells in the gut after exposure to *C. rodentium* and *Salmonella spp*. (38, 39). Additionally, activation of NOD2 receptors by MDP promotes Th17 cells differentiation and activation (40, 41). Denou et al. reported that NOD2 gene deletion increases bacterial invasion of metabolic tissues associated with inflammation and insulin resistance (29). However, whether NOD2 genetic deficiency promotes a dysregulated gut immune environment and precipitates gut dysbiosis-driven metabolic tissue inflammation and T2D onset was not investigated. Recently, our group also demonstrated that IL-17/IL-17R axis drives intestinal neutrophil migration, limits gut dysbiosis, and attenuates LPS translocation to VAT, protecting against metabolic syndrome (42). Additionally, our group also reported that IL-23 deficient mice have reduced intestinal Th17 response, low neutrophil migration, and gut microbiota dysbiosis, resulting in increased susceptibility to obesity-induced metabolic syndrome (43). Our current data identifies NOD2 receptor activation as an upstream pathway in driving the intestinal Th17 response, which reduces LPS translocation, minimizes metabolic inflammation in VAT, and protects against obesity-induced T2D.

In obese and diabetic patients, increased number of Th1 cells associated with reduced Th2 and Treg cell populations occur in the VAT and intestine, compared to healthy individuals (44, 45). A significant increase in gene expression of T-bet transcription factor and IFN-γ cytokine was observed in NOD2^−/−^ mice fed the HFD, when compared to HFD-fed WT mice. In agreement, another study showed that NOD2^−/−^ mice have an exaggerated production of IFN-γ associated with increased number of intestinal Th1 cells (46). It is plausible that the absence of recognition of commensals by the NOD2 receptor leads to a decrease in gut microbiota tolerance mediated by Treg cells and a deviation from the anti to the proinflammatory response in the ileum. A potential consequence of the profound increase in IFN-γ levels in the intestine is a disruption in the intestinal barrier. In fact, IFN-γ decreases the expression of occludin and zonula-1 (ZO-1) in the intestinal epithelium, promoting the breakdown of the intestinal barrier, and the translocation of pathogenic bacteria and their products to the blood and VAT, favoring obesity and insulin resistance (35). Another interesting fact is that IFN-γ decreases insulin sensitivity in enterocytes, the production of mucins and IgA (47). These findings imply that NOD2 activation counteregulates Th1 generation and IFN-γ production in intestinal mucosa and probably favors the maintenance of epithelial barrier during T2D induced by HFD.

Since obesity can lead to changes in commensal bacteria populations, intestinal permeability, and translocation of pathogenic bacteria, we investigated the role of NOD2 in the regulation of gut barrier integrity. Our results show that HFD-fed NOD2^−/−^ mice had significant decrease in expression of occludin and a trend to increase the claudin-2 in the ileum compared to WT mice, indicating a compromised epithelial integrity in these mice. Another study observed that NOD2 is critical for resistance to bacterial infection via expression of intestinal anti-microbial peptides named cryptdins (12). Additionally, NOD2 deficient mice have a reduced number of intestinal intraepithelial lymphocytes, which impairs the integrity of the intestine and leads to altered immune response of the commensal microbiota (48). Also, NOD2 deficiency promotes the dysfunction of Paneth cells and goblet cells in the gut, compromising the production of alpha-defensins and secretion of mucins, which are mucus layer-forming proteins of the intestinal epithelium (49). Our findings demonstrate that NOD2 deficiency contributes to the disruption of the intestinal barrier, serum LPS leakage, which is a possible mechanism that results in metabolic inflammation and insulin resistance worsening in these mice. Overall, our data imply that NOD2 activation reinforces the intestinal barrier function and inhibits LPS translocation, which control insulin resistance and T2D onset induced by HFD.

Considering that the intestinal microbiome is important for food energy extraction, obesity development, and diabetes, we determined the diversity of intestinal bacteria by 16S sequencing. HFD-fed WT mice showed the greatest difference in OTUs richness and an increased relative abundance of the Firmicutes and Proteobacteria phyla, and a reduction in the Verrucomicrobia phylum and the genus *Akkermansia* when compared to the WT mice on control diet. On the other hand, NOD2^−/−^ mice fed the HFD exhibited the lowest OTUs richness and, as expected, a reduction in intestinal microbial diversity. As reviewed by Rinninella et al. (50), a large intestinal bacteria diversity characterizes a healthy gut microbiota composition and both obesity and T2D are associated with changes in the microbial community including lower species diversity and shifts in the relative abundance of some species involved in metabolism. Additionally, an increase in the Bacteroidetes and Verrucomicrobia phyla and *Bacteroides* and *Akkermansia* genera associated to a reduction in the *Lactobacillus* and *Allobaculum genera* were observed in HFD-fed NOD2^−/−^ mice when compared to WT mice fed HFD. Other studies showed that obese mice and humans frequently have an increased abundance of Firmicutes and Proteobacteria, but decreased abundance of Bacteroidetes (51–53). The high abundance of the *Akkermansia muciniphila* bacteria in the gut is related to maintenance of intestinal integrity and reduction of blood glucose levels in humans and rats (54). In addition, *A. muciniphila* regulates the secretion of glucagon-like peptide (GLP-1) by specific cells present in the small intestine and colon, which improves adipocyte metabolism, sensitivity to insulin, and acts as an anti-inflammatory mediator (55). In agreement, increased abundance of Bacteroidetes phylum was also observed in populations of obese individuals and individuals that present propensity to develop obesity and diabetes (56, 57). Therefore, our results imply that Gram-negative *Bacteroides* prevalence over Gram-positive *Lactobacillus* and *Allobaculum* in gut microbiota account for elevated LPS levels in NOD2^−/−^ mice fed the HFD.

**Figure 7:**
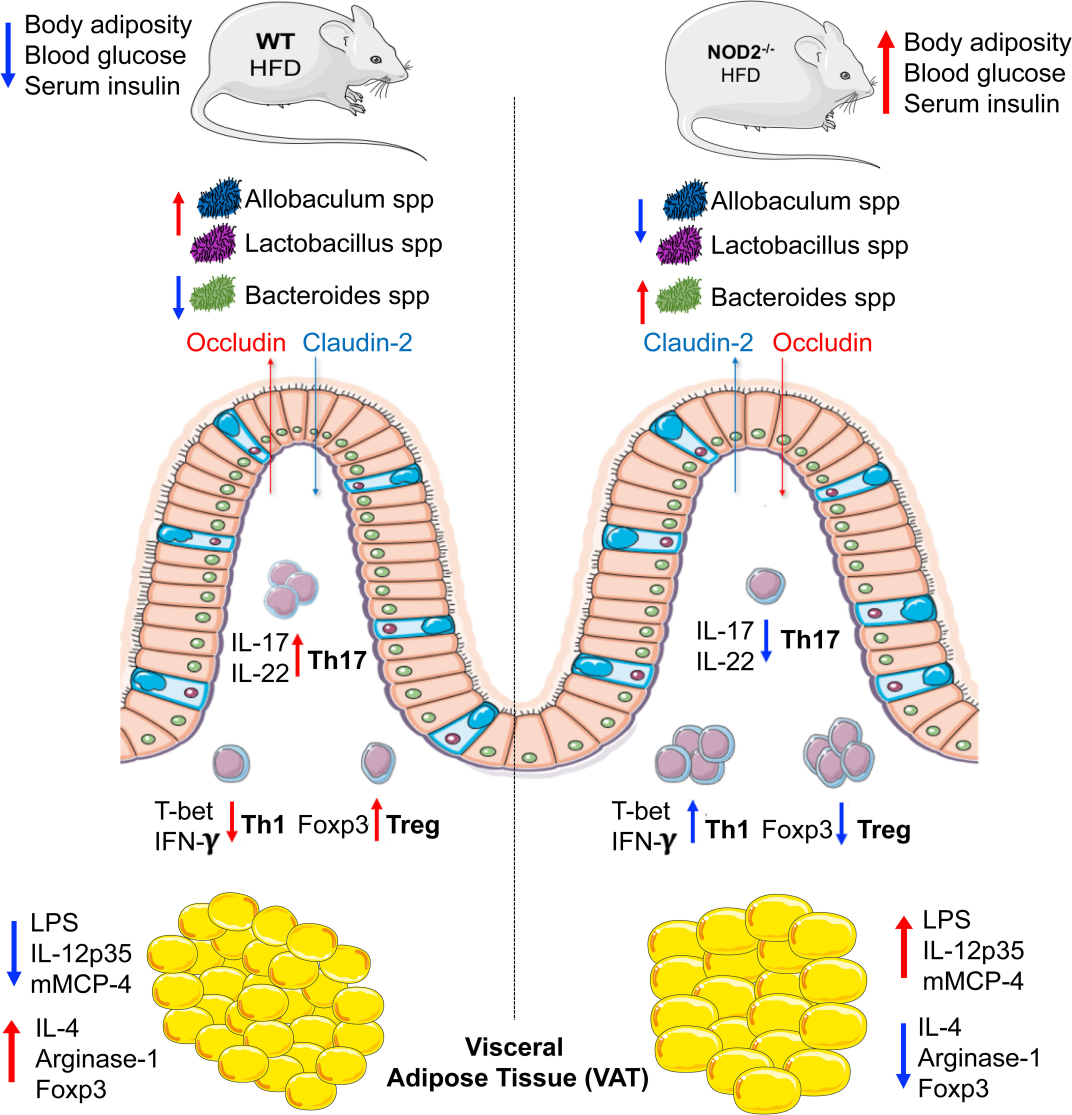
Representation about the immunological mechanisms involved in the protective efffect of NOD2 receptor in the T2D development. Mechanistically, the NOD2 activation drives the intestinal Th17 response and IL-22 and IL-17 expression in the ileum. In turn, promotes the gut homeostasis including a healthy gut microbiota with enrichment of Lactobacillus and Allobaculum genera and decrease of gram-negative Bacteroides genus and maintenance of gut barrier integrity. In addition, a downregulation of the intestinal Th1 response and interferon gamma expression is associated in the ileum. Taken together, these events reduce the gut permeability and endotoxemia, which ultimately favors an anti-inflammatory profile mediated by M2 macrophages in VAT and result in obesity-induced T2D protection.

In summary, we elucidate that NOD2 activation is required for Th17 response induction in the small intestine, which are important to shape a healthy gut microbiota and to maintain the gut barrier integrity. In turn, these adaptive immunological features limit endotoxemia, bacterial translocation, and sustain an anti-inflammatory phenotype mediated by M2 macrophage and Treg in VAT, protecting against obesity-induced metabolic dysfunction and T2D development (Figure 7). Thus, our findings propose the NOD2 receptor as a new therapeutic target in metabolic diseases like obesity, T2D, and their comorbidities.

## Data Availability Statement

The raw data supporting the conclusions of this article will be made available by the authors, without undue reservation, to any qualified researcher.

## Ethics Statement

The experiments were carried out in accordance with the National Council for Animal Experimentation Control (CONCEA) and were approved by the Ethics Committee on Animal Use (CEUA) of the University of São Paulo, Ribeirao Preto, Brazil (protocol number 144/2014).

## Author Contributions

DC designed, performed the experiments and analyzed the results. MP, JL, CP, LM, FR, and TP contributed with the *in vivo* experiments and helped in the revised manuscript. RT edited the manuscript, provided scientific assistance and revised it. VB and TF-S helped with the gut microbiota reanalysis and revised the manuscript. SR supported us with histology and imaging data. JS provided intellectual support in addition to directing and supervising the study. All authors contributed to the article and approved the submitted version.

## Conflict of Interest

The authors declare that the research was conducted in the absence of any commercial or financial relationships that could be construed as a potential conflict of interest.

## Supplementary Material

Supplementary Figure 1 GLUT-4, total and phosphorylated forms of AKT expression in skeletal muscle and histopathological analysis of pancreatic tissues of WT and NOD2^−/−^ mice fed a CTD or HFD. Quantification of GLUT-4, total, or phosphorylated AKT was determined by densitometry in NOD2^−/−^ and WT mice after 20 weeks on HFD or CTD **(A–C)**. Representative images of GLUT-4, total, phosphorylated AKT, or β-actin expression in skeletal muscle **(D)**. Histological analysis of inflammatory infiltrate into pancreatic islets stained with hematoxylin-eosin (H&E) **(E–H)**. The insulin-producing β cell expression was performed into pancreatic islets immunostained with insulin-specific antibody **(I–L)** (original magnification 200x). The results are expressed as the mean ± SEM and are a compilation of 3 independent experiments (*n* = 3–4 mice per group). Asterisks represent statistically significant differences (^*^*p* < 0.05) compared to WT on CTD; (^#^*p* < 0.05) compared to WT on HFD; (^&^*p* < 0.05) compared to NOD2^−/−^ mice on CTD. Significant differences between the groups were compared by one-way ANOVA followed by Tukey's multiple-comparison test.

## Abbreviations

Foxp3: forkhead box P3
HFD: high-fat diet
H_2_O_2_: hydrogen peroxide
IFN-γ: interferon gamma
IL: interleukin
iNOS: inducible nitric oxide synthase
LPS: lipopolysaccharide
MCs: mast cells
MCP-1: monocyte chemoattractant protein-1
MLNs: mesenteric lymph nodes
NOD1: Nucleotide-binding oligomerization domain-containing protein 1
NOD2: Nucleotide-binding oligomerization domain-containing protein 2
PBS: Phosphate buffered saline
PI3K: phosphatidylinositol-4,5-bisphosphate 3-kinase
PMA: phorbol myristate acetate
OTU: operational taxonomic units
SDS: sodium dodecyl sulfate
ROR-γt: RAR-related orphan receptor gamma t
ROS: reactive oxygen species
Th1: Type 1 T helper lymphocytes
Th2: Type 2 T helper lymphocytes
TGF-β: transforming growth factor beta
Th17: IL-17-producing T helper lymphocytes
TLR: toll-like receptors
TNF-α: tumor necrosis factor-α
T2D: type 2 diabetes
Treg: regulatory T lymphocytes
VAT: visceral adipose tissue
WT: wild-type.
