# Supplementary figures and images for: NOD2 Deficiency Promotes Intestinal CD4+ T Lymphocyte Imbalance, Metainflammation, and Aggravates Type 2 Diabetes in Murine Model
Source: Front Immunol. 2020 Jul 7;11:1265. doi: 10.3389/fimmu.2020.01265 (PMC7381387; doi:10.3389/fimmu.2020.01265)

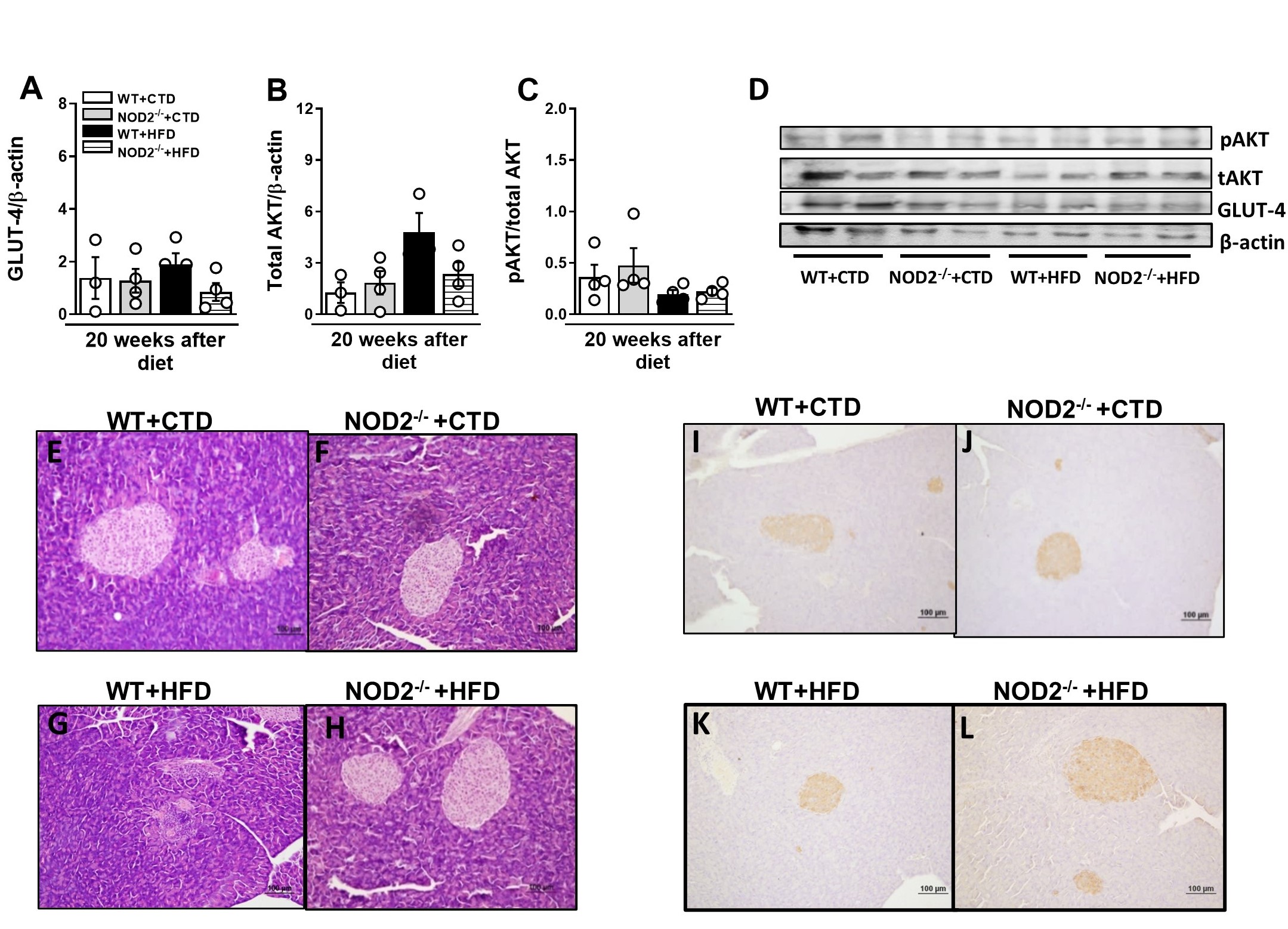

Supplement: Supplementary Figure 1 — GLUT-4, total and phosphorylated forms of AKT expression in skeletal muscle and histopathological analysis of pancreatic tissues of WT and NOD2−/− mice fed a CTD or HFD. Quantification of GLUT-4, total, or phosphorylated AKT was determined by densitometry in NOD2−/− and WT mice after 20 weeks on HFD or CTD (A–C). Representative images of GLUT-4, total, phosphorylated AKT, or β-actin expression in skeletal muscle (D). Histological analysis of inflammatory infiltrate into pancreatic islets stained with hematoxylin-eosin (H&E) (E–H). The insulin-producing β cell expression was performed into pancreatic islets immunostained with insulin-specific antibody (I–L) (original magnification 200x). The results are expressed as the mean ± SEM and are a compilation of 3 independent experiments (n = 3–4 mice per group). Asterisks represent statistically significant differences (*p < 0.05) compared to WT on CTD; (#p < 0.05) compared to WT on HFD; (&p < 0.05) compared to NOD2−/− mice on CTD. Significant differences between the groups were compared by one-way ANOVA followed by Tukey's multiple-comparison test. [file Image_1.jpeg]
